# Case of the Fatal Effect of Opium Obviated

**Published:** 1800-12

**Authors:** 


### *Case of the fatal Effect of Opium obviated.*

On the 30th of September, 1800, I was fent for to fee J. R. a young man about 19 years of age, who, an hour before, had taken an ounce of laudanum, with a view of deftroying himfelf. He was then in a deadly ftupor, and his pulfe 120 in a minute. I ordered him to be ftripped, and directed two men to rub his body and limbs very freely with falt, which was perfevered in, with occafional fhort intervals of reft, for upwards of four hours. As foon as any particular part became hot, or had a tendency to excoriate, I ordered the men to difcontinue the friction on that part. He had already taken a large dofe of ipecacuanha; I afterwards gave him half a drachm of vitriolated zinc, diffolved in one ounce of water. It was got into him in this way: Two tea fpoons full were put into his mouth, he being laid on his back; his nofe was then irritated with a feather dipped in liquor volat. cornu cervi, upon which he was roufed, and deglutition took place. In this way the juice of three lemons was got into him. The emetic did not operate for more than an hour, and at laft vomiting feemed to be effected by irritating the throat with the feather mentioned above. Afterwards he had adminiftered to him, in the fame way, fome wine and water, and a mixture with æther and cordial confection. Stimulating the noftrils roufed him for a few feconds, and made him exert confiderable mufcular ftrength, but he relapfed again immediately into his former ftupor. I ordered his noftrils to be irritated every ten minutes. He took the laudanum at eight o'clock in the evening, and the ftupor did not leave him until two o'clock in the morning. At eight o'clock, A. M. he feemed perfectly recovered, complaining only of forenefs in confequence of the friction.

The indication to be kept in view in obviating the effect of opium, appears to be, as pointed out by Dr. Seaman, to produce fuch a degree of irritation as to counteract its foporific quality. With this intention I prefcribed the emetic, the ftimulation of the noftrils with liq. volat. cornu cervi, and the friction friction with falt. Nothing occurred to me fo likely to produce an equable irritation without fubfequent injury, as friction with falt. The effect it produced fully anfwering my expectation, I gave the lemon juice more in compliance with cuftom, than from any dependance on its antidotal operation. The ftomach is rendered fo torpid by the opium, that very little effect can be produced by the exhibition of internal remedies.

