# Addenda

**Published:** 1800-12

**Authors:** 


### ADDENDA.

To Mr. Veitch's Cafe, p. 513, towards the conclufion of the cafe, and after the words, prefervation of the fcalp, add, "This man was perfectly cured within fix weeks, and difcharged in as full poffeffion (fetting afide the local deprivation of fkull) of his mental and bodily powers as he had been at any time prior to the date of the accident.

"*P. S.* Cow-pox Inoculation is extending itfelf in the Navy, and is much encouraged by the Lords of the Admiralty."

END OF VOL. IV.

