# Errata

**Published:** 1801-01

**Authors:** 


### ERRATA.

Vol. IV. p. 514, l. 12, *for* conical, *read* crucial.

l. 15, *for* infraction, *read* impaction.

l. 35, *for* conically, *read* crucially.

p. 515, l. 39, before meaſurement, place the word *ideal*.

